# Effects of an isocaloric healthy Nordic diet on insulin sensitivity, lipid profile and inflammation markers in metabolic syndrome – a randomized study (SYSDIET)

**DOI:** 10.1111/joim.12044

**Published:** 2013-03-02

**Authors:** M Uusitupa, K Hermansen, M J Savolainen, U Schwab, M Kolehmainen, L Brader, L S Mortensen, L Cloetens, A Johansson-Persson, G Önning, M Landin-Olsson, K-H Herzig, J Hukkanen, F Rosqvist, D Iggman, J Paananen, K J Pulkki, M Siloaho, L Dragsted, T Barri, K Overvad, K E Bach Knudsen, M S Hedemann, P Arner, I Dahlman, G I A Borge, P Baardseth, S M Ulven, I Gunnarsdottir, S Jónsdóttir, I Thorsdottir, M Orešič, K S Poutanen, U Risérus, B Åkesson

**Affiliations:** 1 Institute of Public Health and Clinical Nutrition, University of Eastern FinlandKuopio, Finland; 2 Research Unit, Kuopio University HospitalKuopio, Finland; 3 Department of Medicine and Endocrinology MEA, Aarhus University HospitalAarhus, Denmark; 4 Institute of Clinical Medicine, Department of Internal Medicine, University of OuluOulu, Finland; 5 Institute of Clinical Medicine, Internal Medicine, Kuopio University HospitalKuopio, Finland; 6 Biomedical Nutrition, Pure and Applied Biochemistry, Lund UniversityLund, Sweden; 7 Department of Endocrinology, Skåne University HospitalLund, Sweden; 8 Institute of Biomedicine and Biocenter of Oulu, University of OuluOulu; 9 Department of Psychiatry, Kuopio University HospitalKuopio, Finland; 10 Department of Public Health and Caring Sciences, Clinical Nutrition and Metabolism, Uppsala UniversityUppsala, Sweden; 11 Center for Clinical Research DalarnaFalun, Sweden; 12 Eastern Finland Laboratory Centre and Department of Clinical Chemistry, University of Eastern FinlandKuopio, Finland; 13 Institute of Clinical Medicine, University of Eastern FinlandKuopio, Finland; 14 Department of Nutrition, Exercise and Sport, University of CopenhagenCopenhagen, Denmark; 15 Department of Epidemiology, School of Public Health, Aarhus UniversityAarhus, Denmark; 16 Department of Cardiology, Aalborg Hospital, Aarhus University HospitalAarhus, Denmark; 17 Department of Animal Science, Aarhus UniversityAarhus, Denmark; 18 Department of Medicine (H7), Karolinska InstituteStockholm, Sweden; 19 Nofima, Norwegian Institute of Food, Fisheries and Aquaculture ResearchÅs, Norway; 20 Department of Health, Nutrition and Management, Faculty of Health Sciences, Oslo and Akershus University College of Applied SciencesOslo, Norway; 21 Unit for Nutrition Research, University of Iceland and Landspitali – The National University Hospital of IcelandReykjavik, Iceland; 22 VTT Technical Research Centre of FinlandEspoo, Finland; 23 Department of Clinical Nutrition, Skåne University HospitalLund, Sweden

**Keywords:** cardiovascular risk, inflammation, intervention, metabolic syndrome, Nordic diet

## Abstract

**Background:**

Different healthy food patterns may modify cardiometabolic risk. We investigated the effects of an isocaloric healthy Nordic diet on insulin sensitivity, lipid profile, blood pressure and inflammatory markers in people with metabolic syndrome.

**Methods:**

We conducted a randomized dietary study lasting for 18–24 weeks in individuals with features of metabolic syndrome (mean age 55 years, BMI 31.6 kg m^−2^, 67% women). Altogether 309 individuals were screened, 200 started the intervention after 4-week run-in period, and 96 (proportion of dropouts 7.9%) and 70 individuals (dropouts 27%) completed the study, in the Healthy diet and Control diet groups, respectively. Healthy diet included whole-grain products, berries, fruits and vegetables, rapeseed oil, three fish meals per week and low-fat dairy products. An average Nordic diet served as a Control diet. Compliance was monitored by repeated 4-day food diaries and fatty acid composition of serum phospholipids.

**Results:**

Body weight remained stable, and no significant changes were observed in insulin sensitivity or blood pressure. Significant changes between the groups were found in non-HDL cholesterol (−0.18, mmol L^−1^ 95% CI −0.35; −0.01, *P* = 0.04), LDL to HDL cholesterol (−0.15, −0.28; −0.00, *P* = 0.046) and apolipoprotein B to apolipoprotein A1 ratios (−0.04, −0.07; −0.00, *P* = 0.025) favouring the Healthy diet. IL-1 Ra increased during the Control diet (difference −84, −133; −37 ng L^−1^, *P* = 0.00053). Intakes of saturated fats (E%, beta estimate 4.28, 0.02; 8.53, *P* = 0.049) and magnesium (mg, −0.23, −0.41; −0.05, *P* = 0.012) were associated with IL-1 Ra.

**Conclusions:**

Healthy Nordic diet improved lipid profile and had a beneficial effect on low-grade inflammation.

## Introduction

Diet has a great impact on the risk and worldwide burden of chronic diseases, including cardiovascular diseases (CVD), the metabolic syndrome (MetS) and type 2 diabetes 1, 2. In particular, the effects of dietary fatty acids on the metabolism of serum lipids and lipoproteins and their connection to atherosclerotic process have been thoroughly investigated 3, 4, whereas their effects on low HDL cholesterol 5–8, insulin resistance and low-grade inflammation are still unclear 9, 10. MetS, characterized by insulin resistance, central obesity, clustering of cardiometabolic risk factors and low-grade inflammation, is a major clinical risk indicator of type 2 diabetes and CVD globally 1, 2. The Mediterranean diet, representing the diet traditionally eaten in Southern Europe, has long been related to improved health and prevention of CVD, certain cancers and type 2 diabetes 11–18. Acceptance of the Mediterranean diet has not been easy in other parts of the Western world, probably due to difficulties in changing dietary patterns, cultural differences in taste and limited accessibility to various foods 19, 20. A health-enhancing regional Nordic diet has therefore been proposed as an alternative to the Mediterranean diet 21. Recent studies suggest that a healthy Nordic diet pattern is related to lower mortality 22 and improved cardiovascular risk factors in short term 23. The aim of the present study was to clarify whether a Nordic alternative for healthy food pattern in a weight-stable condition would have beneficial effects on insulin resistance, glucose tolerance, serum lipids and lipoproteins, and inflammatory markers in people with MetS. The duration of our trial was decided to last for 18–24 weeks to monitor adherence to the diet and analyse the longer-term effects on glucose tolerance, insulin sensitivity and HDL-C metabolism, because there is evidence that short-term results may differ from that achieved in trials with longer duration 24.

## Materials and methods

### Study design

**Fig. 1:**
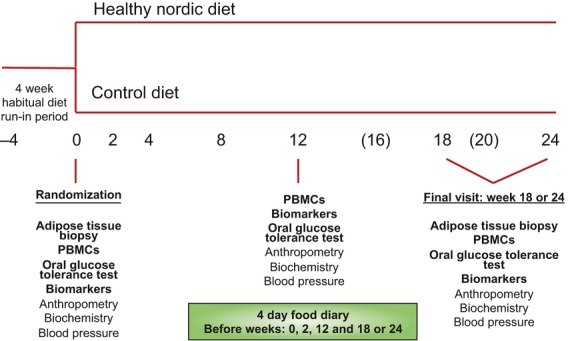
Study design in the Healthy Nordic diet intervention study. In four centres, the main outcome measurements were taken at week 18. PBMCs = peripheral blood mononuclear cells.

The study was a randomized controlled multicentre study performed in six centres [Kuopio and Oulu (Finland), Lund and Uppsala (Sweden), Aarhus (Denmark) and Reykjavik (Iceland)). The primary outcome was insulin sensitivity and glucose tolerance, and secondary outcomes were blood lipids, blood pressure and inflammatory markers as they are all closely related to insulin resistance and are risk factors for CVD. The study design and the main measurements at each time-points are described in Fig. 1. There was 4-week run-in period during which all participants followed their habitual diet before the participants, fulfilling the inclusion criteria, were randomized into a control (Control diet) group or a Healthy Nordic diet group (Healthy diet) for the next 18–24 weeks. Randomization was performed by matching according to gender and medians of age, body mass index (BMI) and fasting plasma glucose at screening, resulting in equal amounts of certain strata classes amongst the groups. The study participants were planned to visit the study clinic at 2, 4, 8, 12, 16, 20 and 24 weeks. The major visits were in the beginning (0 week) and at 12 and at either 18 or 24 weeks (end of the study). The staff in all study centres were trained to perform the measurements in a similar way, and a common quality management protocol was made familiar to all staff members at each study site. The original protocol was changed after a consultation with the NordForsk Panel and Scientific Committee members after the trial had been started for the following reasons: (i) a shorter period of intervention (i.e. 18 weeks) was considered to give the same information as that obtained from 24 weeks' trial, (ii) total costs would be reduced, and (iii) recruitment of study subjects was easier for a shorter trial. Therefore, it was decided to shorten the intervention to 18 (+/− 1) weeks in four centres (Aarhus, Uppsala, Reykjavik and Oulu), whereas in Lund and Kuopio where the intervention was started earlier, the original study design was followed. In Kuopio and Lund, the intervention was carried out from October 2009 to June 2010, in Aarhus, from January 2010 to September 2010, in Oulu, from December 2009 to October 2010, in Reykjavik, from March 2010 to October 2010 and in Uppsala, from June 2010 to November 2010. The visits to the study centres were in line with the original study plan, and the study was strictly blinded regarding the measurements until the trial was completed in all centres. Thus, in four centres with a shorter intervention (18 weeks), the final measurements according to the original study plan were taken at 18 weeks. The study participants were advised to keep weight and physical activity constant and not to change their smoking and drinking habits or drug treatment during the study.

All study participants provided their written informed consent, and local Ethical committees of all the participating centres approved the study protocol.

#### Screening of study participants and inclusion and exclusion criteria

A screening examination was carried out 4 weeks before the start of the dietary intervention. This visit included medical history and a clinical examination, including ECG (if deemed necessary by the study physician), body weight, height, waist circumference, blood pressure, blood count, fasting plasma glucose (screening value < 7 mmol L^−1^) and fasting serum creatinine, thyroid-stimulating hormone (TSH), liver enzymes (gamma-glutamyltransferase, alanine aminotransferase, alkaline phosphatase), serum triglycerides, total cholesterol and HDL cholesterol. The inclusion criteria were age 30–65 years, BMI 27–38 kg m^−2^ and two other of IDF's criteria for MetS 2, fasting plasma glucose ≤7.0 mmol L^−1^ and a 2-h glucose value < 11.1 mmol L^−1^ at baseline detected by an oral glucose tolerance test. As the applied IDF criteria 2 concern the white population, only whites were included in the study. Antihypertensive and lipid-lowering medication was allowed but without dosage changes during the trial. The main exclusion criteria included any chronic disease and condition, which could hamper the adherence to the dietary intervention protocol, poor compliance, chronic liver, thyroid and kidney diseases, alcohol abuse (>40 g per day), diabetes, fasting triglycerides >3.0 mmol L^−1^, total cholesterol >6.5 mmol L^−1^ and blood pressure >160/100 mmHg. A few study participants with triglycerides between 3 and <4 mmol L^−1^ and with BMI between 38 and <40 kg m^−2^ were, however, included due to the fact that they were very keen to take part in ongoing the trial. Other exclusion criteria were a recent myocardial infarction (<6 months), corticosteroid therapy, psychiatric disorders needing drug treatment, ongoing or recent treatment for cancer, coeliac disease, allergies to cereals or fish and other serious and extensive food allergies. Exceptional diets were also an exclusion criterion as well as binge eating 25.

Inhaled corticosteroids were permitted. Participants taking fish and vegetable oil supplements or using stanol or sterol esters were asked to discontinue using these supplements at least 4 weeks before the beginning of the intervention. None of the study participants were excluded due to noncompliance with regard to the study protocol. The allowed weight change during the study was less than 4 kg.

#### Study participants

**Fig. 2:**
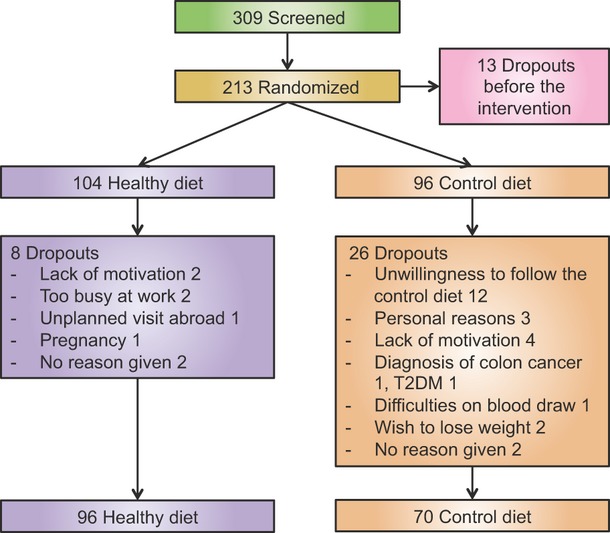
Flow chart and the number of participants and dropouts from the study.

Participants were mainly recruited through advertising in newspapers, but also from previous clinical or epidemiological trials. Altogether 309 individuals were originally contacted and screened, and they visited the study clinics as shown in the flow chart (Fig. 2). After initial exclusions, 213 of them were randomized, but there were 13 additional dropouts before the intervention. Thus, 200 individuals started the intervention, 104 in the Healthy diet group and 96 in the Control diet group. There were eight additional dropouts (five women and three men) in the Healthy diet group and 26 (20 women and 6 men) in the Control diet group during the study, and altogether 96 individuals in the Healthy diet group and 70 in the Control diet group completed the trial. According to the original study design, altogether 11 participants (five in Healthy diet and six in Control diet groups, respectively) were excluded from final analyses due to weight change of over 4 kg. Reasons for dropouts are given in the flow chart (Fig. 2), but we also analysed the data without any exclusions.

#### Study diets and dietary counselling

The nutrient composition of the diets is summarized in Table S1. Nordic nutrition recommendations formed the basis for the Healthy diet 26, and the mean nutrient intake in the Nordic countries formed the basis for the Control diet. The main differences between the diets were the amount of dietary fibre and salt, and the quality of dietary fat. Both the Healthy diet and the Control diet were isocaloric based on the evaluation of the habitual diet (calculated from a 4-day food record) during the run-in period. National nutrient databases were used to calculate energy, macronutrient, cholesterol, fibre and micronutrient contents [Aivo Finland Ltd, Turku, based on the database of the National Institute of Health and Welfare, Finland, Dietist XP Software Package, version 3.1 (2009) linked to Swedish Food Database 2009, Sweden, Master Dietist System version 1.235 (2007) based on Danish National Food administration database, Denmark, and The Icelandic Food Composition Database (ISGEM), Iceland].

In the Healthy Nordic diet, the main emphasis was on food items such as whole-grain products, abundant use of berries, fruit and vegetables, rapeseed oil, three fish meals per week, low-fat dairy products and avoidance of sugar-sweetened products (Table 1). Key products were provided to the study participants in both groups. The participants in the Healthy Nordic diet group received whole-grain products, for example, various breads, berry products as frozen berries (e.g. strawberries, black currants, bilberries) and dried powder as well as dietary fats including rapeseed oil- and vegetable oil-based spreads. Local fruits (not provided) were mostly apples, pears and plums. Furthermore, either fish was directly provided or the expenses for consuming fish were covered to the study participants. The individuals in the Control diet group received low-fibre cereal products, for example, breads with fibre content <6 g per 100 g, and dairy fat-based spread, for example butter.

**Table 1:** Food items recommended in the study diets

Food group	Healthy Nordic diet	Control diet
Cereal products	≥25% of total energy as whole grain of which ≥50% as rye, barley and oat. Whole-grain pasta and unpolished rice (≥6 g fibre per 100 g) ≥2–3 meals per week. Low salt content (≤1.0%) in breads. Cereals with no added sugar or honey. Bread (≥6 g fibre per 100 g) ≥6 slices per day	≥25% of energy as refined of which ≥90% as wheat
Vegetables, berries, fruit (potatoes not included)	Fruits, vegetables and berries ≥500 g of which berries ≥150–200 g per day, fruit ≥175 g per day and vegetables ≥175 g per day	200–250 g, no bilberries
Dietary fats	Rapeseed oil. Rapeseed and/or sunflower oil and/or soya bean oil-based margarines with no trans fatty acids and ≥2/3 of unsaturated fats (unsalted nuts and seeds can be included in the diet)	Butter or other milk fat-based spread (≥50% of total fat as saturated)
Dairy products	Low-fat liquid dairy ≤1% of fat Cheese ≤17% of fat, ≥2 portions per day. Sweetened yoghurts and other fruit milk products avoided.	No limitations
Fish	≥3 meals per week, 2 fatty fish (>4–5% of fat) meals + 1 low-fat fish meal.	≤1 meal per week
Meat	Preferably white meat, poultry. Low-fat choices. Game.	No limitations
Beverages	Avoidance of sugar-sweetened beverages. Fruit and berry juices <1 glass (1.5 dL) per day	No limitations

A clinical nutritionist or a dietician instructed the diets at the 0-week visit. The study participants kept dietary records regarding the intake of cereal products, berry products, vegetables and fish. These records were checked at each visit to the study centre, including the visits when the groceries were delivered to the participants, that is, at 1–2-week intervals. In addition to the 4-day food record during the run-in period, the participants kept 4-day food records also at weeks 2, 11 and 17 or 23 before the next visit to the study centre for calculations of the dietary intake during the intervention. All food records included four consecutive days of which one was a weekend day.

Physical activity (total, leisure time, commuting and at work) was monitored by a questionnaire. The aim was to keep physical activity unchanged.

#### Biochemical and anthropometric measurements

Screening laboratory measurements, glucose and lipid values and anthropometric measurements were performed locally according to the standard operational procedures agreed by all centres. Centralized analyses were as follows: apolipoproteins A1 (Apo A1) and B (Apo B), cytokines and adipokines (University of Eastern Finland and Kuopio University Hospital), fatty acid composition of serum phospholipids (Uppsala University), 24-h urine sodium and potassium excretion (Copenhagen University) and plasma insulin (Aarhus University Hospital).

A standard 2-h oral glucose tolerance test (75 g d-glucose) was performed after 12-h fast in the morning. Blood samples were taken at the time-points 0, 30 and 120 min to measure the concentrations of glucose and insulin.

Automated clinical chemistry analysers and routine clinical chemistry methods were used to measure glucose, cholesterol, triglycerides, HDL-C, Apo A1 and Apo B, high-sensitive C-reactive protein (hs-CRP) and the liver enzyme activities. For these automated analyses, the equipment and system reagents were from Siemens Healthcare Diagnostics (Tarrytown, NY, USA), Thermo Fisher Scientific (Vantaa, Finland), Roche Diagnostics GmbH (Mannheim, Germany) (in two laboratories), Toshiba (Japan), Abbott Laboratories (Irving, TX, USA), Ortho-Clinical Diagnostics and Johnson & Johnson (New Brunswick, NJ, USA). The sample material was either plasma or serum as required by the analytical method in each laboratory. The within-run variations (CV%) for the biochemical measurements were 0.4–4.6%. The between-run CV% was 0.5–6%. LDL-C concentrations were calculated using Friedewald's formula. Plasma insulin was measured using the ELISA method from Dako Denmark A/S, Glostrup, Denmark, with a within-run and between-run CV% <8% and <10%, respectively. Na^+^ and K^+^ were measured in 24-h urine samples on a Cobas Fara analyser equipped with ion-selective electrodes (Roche). The CV% for six intrabatch identical samples was <2%. The 24-h excretion of each ion was calculated by multiplying the concentration by the volume excreted.

The plasma interleukins (IL) IL-1 beta, IL-1 Ra, IL-6, IL-10 and tumour necrosis factor receptor II (TNF RII) assays were performed using ELISA methods from R&D Systems Inc, Minneapolis, MN, USA. The within- and between-run variations (CV%) were lower than 14%, except for IL-10 and TNF RII, for which they were 10−30%. Some high values or individuals with greatly elevated (>2 SD) inflammatory marker values were excluded from statistical analyses (IL-1 Ra >1500 ng L^−1^, three values, IL-1 beta >4 ng L^−1^, five values, two individuals, IL-6 >6 ng L^−1^, eight values, one individual, IL-10 >5 ng L^−1^, five values, two individuals, TNF RII >3000 ng L^−1^, five values, hs-CRP >10 mg L^−1^, eight values, one individual, and in this case also, IL-6 values were excluded]. Therefore, the exact numbers for each measurement vary in the results given in the tables.

Serum high molecular weight (HMW) adiponectin was measured using ELISA kit from Millipore (St Charles, MO, USA) after the specific proteolytic digestion of other multimeric adiponectin forms (LMW, MMW adiponectin). The within-run CV% was 2–6%, and the between-run CV was 12%.

Phospholipid fatty acid composition was analysed as previously described in detail 27. The CV varied between 0.2% and 5% in successive gas chromatography runs. The relative amount of fatty acids is expressed as the percentage of the total amount of identified fatty acids.

Indices for insulin sensitivity and secretion and area under the curve (AUC) values for glucose and insulin were calculated 28, 29.

### Statistical analysis

Based on our experiences from previous clinical trials 23, 29 and power calculations (alpha <0.05, beta >0.8) on serum cholesterol, fasting glucose and insulin, 80–90 subjects in each group were expected to provide sufficient statistical power. The primary analyses were performed using linear mixed-effects models (nlme package version 3.1–102) 30. Models were fit using a restricted maximum likelihood (REML) method whilst ignoring missing observations. The age variable was log_10_-transformed prior to analyses to address the skewed distribution. The models included the outcome of interest as dependent variable, subject identifier as a random effect and body weight, age, gender, study centre (i.e. also study duration), study group, time-point and study group * time-point interaction as covariates. Analyses of systolic and diastolic blood pressures included antihypertensive treatment as covariate, and analyses of lipids and inflammation markers included statin usage as a covariate. Subgroup analyses of lipids and inflammation markers were also performed excluding statin users. The aim of the analyses was to study the effect of study group on the outcome of interest during the intervention, that is, the study group * time-point (baseline, 12 weeks and/or end of the intervention) interaction. An additional analysis was performed to study the association of IL-1 Ra with selected dietary variables within baseline, week 12 and weeks 18/24 measurements. The models included IL-1 Ra level as dependent variable, subject identifier as a random effect and body weight, age, gender, study centre and statin usage as covariates. All analyses were performed using R version 2.14 (R Development Core Team, 2011) 31. We also performed statistical analyses without applying any exclusion criterion, and the results remained quite similar. Furthermore, between-group differences in the main outcome measurements were tested by *t*-test without any adjustment, and the results were similar except for non-HDL cholesterol (see the Results section).

## Results

### Baseline characteristics

Table 2 shows the baseline characteristics by group. The number of women was 63% in the Control diet group and 70% in the Healthy diet group. There were no significant differences between these two groups in BMI, age, serum lipids, Apo A1 or Apo B, glucose tolerance, blood pressure, smoking, statin use or antihypertensive drug treatment, or the prevalence of MetS (92% in Control and 91% in the Healthy diet group).

**Table 2:** Baseline characteristics^a^

	Healthy diet (*n* = 99)	Control diet (*n* = 90)
Sex (female)	69 (70%)	57 (63%)
Age (years)	54.0 (8.5)	54.9 (8.6)
BMI (kg m^−2^)	31.6 (3.5)	31.7 (2.8)
Total cholesterol (mmol L^−1^)	5.30 (0.88)	5.24 (0.98)
LDL cholesterol (mmol L^−1^)	3.25 (0.80)	3.21 (0.89)
HDL cholesterol (mmol L^−1^)	1.36 (0.33)	1.33 (0.41)
Triglycerides (mmol L^−1^)	1.52 (0.75)	1.61 (0.80)
Apo B (g L^−1^)	1.06 (0.26)	1.05 (0.26)
Apo A–I (g L^−1^)	1.44 (0.20)	1.40 (0.24)
0 h Glucose (mmol L^−1^)	5.8 (0.6)	5.7 (0.6)
2 h Glucose (mmol L^−1^)	6.2 (1.6)	6.8 (2.1)
Systolic BP (mmHg)	130 (15)	130 (16)
Diastolic BP (mmHg)	82 (10)	82 (11)
Statin use	18 (18%)	26 (29%)
Smoking	4 (4%)	9 (10%)
Drug treatment for hypertension	56 (57%)	42 (47%)
Metabolic syndrome	91 (92%)	78 (87%)

Values are given as means (SD) or numbers (*N*, %).

a Participants with body weight change over 4 kg were excluded due to the study design (five in the Healthy diet and six in the Control diet groups, respectively).

### Dietary data

At baseline, no major differences were found in dietary variables between the groups (Table 3). There were significant differences between the groups during the intervention in the intakes of carbohydrates, protein, total fat, saturated fatty acids, polyunsaturated fatty acids, alpha-linolenic acid, fibre, dietary cholesterol, salt and sodium, beta-carotene, vitamin C, vitamin E, potassium and magnesium, generally favouring the adherence to the Healthy Nordic diet. As for the major food items (total fat, saturated fats, monounsaturated and polyunsaturated fatty acids and dietary fibre), the goals of the Healthy diet were satisfactorily achieved (Supplemental Table S1). There was no significant difference between the groups in the reported alcohol intake at baseline, and the changes in alcohol intakes were quite modest during the trial (Table 3). No significant differences were found in the mean values (before and during the intervention) or changes in 24-h sodium and potassium excretion between the groups in the subgroups of individuals with reliable urine collection (*N* = 84 for Healthy diet group and *N* = 62 for Control diet group). The figures [mean (SD)] for 24-h urinary sodium and potassium excretions (mmol per day) before and during the trial (12-week and 18- to 24-week collections combined) were as follows: 143.5 (51.5) and 141.5 (64.9) for sodium and 66.7 (65.3) and 65.3 (22.5) for potassium in the Healthy diet group and 150.7 (72.4) and 148.2 (60.0) for sodium and 69.4 (24.8) and 71.1 (24.7) for potassium in the Control diet group, respectively, and the changes in sodium and potassium excretions between the groups (1.8 mmol per day (−16.7; 20.2, *P* = 0.85) and −2.5 (−9.1; 4.1, *P* = 0.46) were negligible.

**Table 3:** Dietary data at baseline and during the study (2-, 12- and 18/24-week data (mean and SD) by group, and the differences in dietary variables at the end of the study with 95% CI

	Healthy diet (0 week)	Healthy diet (2, 12, 18/24 week)	Control diet (0 week)	Control diet (2, 12, 18/24 week)	Estimate (95% CI)	Significance (*P*-value)
Energy (kJ)	8523 (1976)	8545 (1812)	8429 (2050)	8540 (1886)	−77 (−496 to 342)	0.72
Carbohydrate (E%)	45.7 (6.0)	46.8 (6.4)	46.1 (6.7)	44.6 (6.5)	2.66 (0.94 to 4.37)	0.0024
Sucrose (g)	39.6 (23.6)	34.7 (18.4)	38.7 (17.6)	35.7 (19.8)	−2.16 (−7.19 to 2.86)	0.4
Protein (E%)	16.6 (2.8)	17.5 (3.2)	16.9 (2.3)	16.2 (2.3)	1.51 (0.69 to 2.33)	0.00033
Fat (E%)	33.2 (6.3)	31.7 (5.3)	32.9 (5.9)	35.2 (5.2)	−3.63 (−5.28 to −1.98)	1.90E-05
SFA (E%)	12.6 (3.3)	10.1 (2.5)	13.1 (3.1)	14.8 (2.9)	−4.3 (−5.1 to −3.4)	3.70E-21
MUFA (E%)	11.3 (2.7)	11.6 (2.5)	11.1 (2.3)	12.1 (2.2)	−0.7 (−1.3 to 0.0)	0.058
PUFA (E%)	4.9 (1.7)	6.8 (2.0)	4.6 (1.4)	4.4 (1.2)	2.1 (1.6 to 2.6)	3.30E-16
Linoleic acid (g)	8.2 (3.6)	8.8 (4.7)	7.2 (2.4)	8.0 (3.0)	−0.1 (−1.3 to 1.1)	0.89
Alpha-linoleic acid (g)	1.5 (1.6)	2.0 (1.8)	1.2 (0.7)	1.3 (0.6)	0.5 (0.2 to 0.8)	0.0017
Fibre (g)	22.4 (7.1)	34.7 (10.2)	20.3 (6.1)	15.9 (4.5)	16.8 (14.8 to 18.9)	2.50E-45
Cholesterol (mg)	284 (125)	248 (116)	285 (114)	297 (116)	−45 (−79 to −11)	0.01
Salt (g)	7.4 (2.2)	6.5 (2.2)	7.2 (2.5)	7.1 (2.3)	−0.80 (−1.40 to −0.20)	0.0094
Beta-carotene (μg)	3254 (3067)	4229 (4126)	2775 (3151)	1922.4 (1682)	1882 (987 to 2778)	4.30E-05
Vitamin C (mg)	117 (64)	164 (73)	106 (81)	79 (57)	75 (56 to 94)	7.00E-14
Vitamin E (mg)	9.2 (3.5)	12.1 (4.0)	8.3 (3.0)	7.8 (2.8)	3.5 (2.5 to 4.4)	1.40E-11
Folate (μg)	322 (260)	351 (146)	253 (76)	251 (98)	33 (−13 to 79)	0.16
Sodium (mg)	2945 (881)	2609 (889)	2848 (977)	2817 (863)	−293 (−523 to −63)	0.013
Potassium (mg)	3468 (933)	3793 (972)	3419 (1083)	2938 (886)	796 (593 to 1000)	9.60E-14
Magnesium (mg)	351 (97)	398 (101)	336 (100)	290 (82)	93 (72 to 113)	1.60E-17
Alcohol (E%)	3.0 (4.1)	2.3 (3.5)	2.8 (3.9)	3.3 (4.2)	−1.21 (−2.09 to −0.32)	0.0075

Total physical activity and leisure time physical activity remained unchanged during the study (data not shown).

### Fatty acid composition of phospholipids

Table S2 shows the results of the fatty acid composition. The main differences reflect the different intakes of milk fat between the groups, for example, myristic acid (*P* = 0.08) and especially pentadecanoic acid proportions (*P* = 0.00024) decreased in the Healthy diet group compared with the Control diet group. In addition, the changes in oleic (*P* = 0.039) acid and alpha-linolenic acids (*P* = 0.076) were different between the groups, the latter reflecting the higher intake of rapeseed oil in the Healthy diet group. Changes in dihomo-gamma-linolenic acid proportions were markedly different (*P* = 0.00021) with a decrease in the Healthy diet group and an increase in the Control diet group. Proportions of eicosapentaenoic acid (EPA) and docosahexaenoic acid (DHA) increased in the Healthy diet group and decreased in the Control diet group (between-group difference; *P* < 0.0001 for both fatty acids).

### Body weight, glucose tolerance and liver enzymes

There were no significant within-group changes in body weight, and importantly, the groups did not differ with regard to the mean change in body weight during the trial. There were no differences in any of the variables reflecting glucose metabolism or blood pressure at the end of the trial, similarly serum liver enzyme activities did not change significantly during the intervention (Table 4).

**Table 4:** Clinical and biochemical values at baseline and at the end of the study (mean and SD) by group, and the differences in changes with 95% CI

	Healthy diet (*N* = 88–99)		Control diet (*N* = 64–90)		Estimate (95% CI)	*P*-value
	
	Baseline mean (SD)	End mean (SD)	Baseline mean (SD)	End mean (SD)		
Body weight, kg	90.1 (13.3)	90.4 (13.9)	91.9 (12.6)	92.9 (13.1)	−0.5 (−1.0 to 0.1)	0.097
Total cholesterol, mmol L^−1^	5.30 (0.88)	5.16 (0.92)	5.24 (0.98)	5.17 (0.97)	−0.12 (−0.30 to 0.07)	0.21
LDL cholesterol, mmol L^−1^	3.25 (0.80)	3.12 (0.79)	3.21 (0.89)	3.20 (0.88)	−0.15 (−0.31 to 0.01)	0.058
HDL cholesterol, mmol L^−1^	1.36 (0.33)	1.41 (0.37)	1.33 (0.41)	1.32 (0.37)	0.05 (−0.01 to 0.11)	0.074
Non-HDL cholesterol, mmol L^−1^	3.93 (0.91)	3.71 (1.00)	3.86 (1.05)	3.80 (1.06)	−0.18 (−0.35 to −0.01)	0.04
Triglyceride, mmol L^−1^	1.52 (0.75)	1.41 (0.66)	1.61 (0.80)	1.46 (0.49)	−0.03 (−0.18 to 0.11)	0.64
Apo B, g L^−1^	1.06 (0.26)	1.04 (0.27)	1.05 (0.26)	1.06 (0.25)	−0.04 (−0.08 to 0.00)	0.081
Apo A1, g L^−1^	1.44 (0.20)	1.47 (0.26)	1.40 (0.24)	1.40 (0.23)	0.03 (−0.01 to 0.08)	0.19
LDL-C/HDL-C ratio	2.53 (0.89)	2.38 (0.89)	2.63 (1.10)	2.61 (0.98)	−0.15 (−0.29 to −0.00)	0.046
Apo B/Apo A1 ratio	0.76 (0.22)	0.73 (0.22)	0.77 (0.23)	0.78 (0.22)	−0.04 (−0.07 to −0.00)	0.025
IL-1 Ra, ng L^−1^	366 (217)	364 (194)	345 (163)	441 (262)	−84 (−130 to −37)	0.00053
IL-1 beta, ng L^−1^	0.20 (0.27)	0.23 (0.44)	0.26 (0.37)	0.25 (0.36)	0.05 (−0.07 to 0.18)	0.42
IL-6, ng L^−1^	1.53 (0.71)	1.69 (1.06)	1.51 (0.82)	1.59 (0.92)	0.12 (−0.18 to 0.42)	0.44
IL-10, ng L^−1^	1.16 (0.68)	1.20 (0.75)	1.16 (0.57)	1.23 (0.72)	−0.01 (−0.22 to 0.21)	0.96
sTNFRII, ng L^−1^	1936 (437)	1911 (421)	1911 (407)	1923 (381)	−39 (−138 to 62)	0.45
hsCRP, mg L^−1^	2.6 (2.3)	2.7 (2.2)	2.4 (2.0)	2.3 (1.9)	0.3 (−0.2 to 0.8)	0.18
HMW adiponectin, μg L^−1^	5.43 (3.48)	5.63 (3.27)	4.72 (3.32)	4.68 (3.12)	0.05 (−0.37 to 0.47)	0.81
0 h Glucose, mmol L^−1^	5.75 (0.60)	5.67 (0.65)	5.70 (0.62)	5.58 (0.74)	−0.02 (−0.15 to 0.12)	0.79
2 h Glucose, mmol L^−1^	6.15 (1.57)	6.47 (1.97)	6.78 (2.14)	6.69 (2.11)	0.36 (−0.08 to 0.79)	0.11
InsAUC0-30/GluAUC0-30	25.59 (13.70)	25.62 (11.57)	29.16 (18.12)	30.56 (17.33)	−1.13 (−4.10 to 1.85)	0.46
Matsuda Insulin Sensitivity Index	6.23 (3.34)	6.27 (3.92)	5.65 (3.61)	6.31 (4.76)	−0.25 (−1.13 to 0.63)	0.58
Systolic BP, mmHg	129.9 (14.6)	126.2 (14.6)	129.8 (16.0)	127.9 (15.0)	−2.0 (−5.7 to 1.66)	0.28
Diastolic BP, mmHg	82.5 (10.3)	79.7 (10.5)	81.8 (10.9)	79.2 (10.0)	−1.2 (−3.4 to 1.0)	0.29
Gamma-glutamyltransferase, U/L	37.17 (28.76)	38.63 (31.47)	34.75 (21.71)	37.58 (28.07)	−1.63 (−6.57 to 3.32)	0.52

### Serum lipids and apolipoproteins

**Fig. 3:**
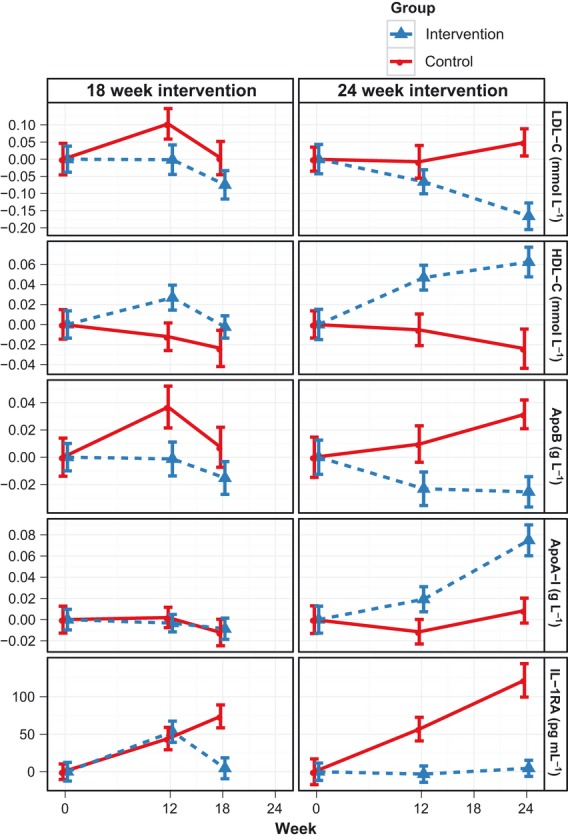
Changes in serum LDL-C, HDL-C, Apo B, Apo-A1, and IL-1 Ra in the SYSDIET study by group (solid line for Control and dotted line for Healthy diet group). The results are given separately for centres with 18 and 24 weeks of the duration of the study.

There were significant decreases in non-HDL-C (adjusted *P* = 0.04, *t*-test *P* = 0.20) and nonsignificant trends towards differences between the groups in changes of LDL-C (*P* = 0.06), Apo B (*P* = 0.08) and HDL-C (*P* = 0.074). Furthermore, LDL-C to HDL-C and Apo B to Apo A1 ratios decreased in the Healthy diet group compared with the Control diet group. When statin users were excluded, the changes in lipid and lipoprotein values were numerically quite similar to that seen in the entire study population (data not shown). Figure 3 depicts the changes in LDL-C, HDL-C and Apo B and Apo A1 with time separately for those with 18- and 24-week follow-up. It is clear that the duration of the study affects the trends and suggests some heterogeneity in changes across the centres.

### Inflammatory markers

The IL-1 Ra level increased in the entire Control diet group (−84; −130 to −37 ng L^−1^; group difference comparing the Healthy diet to Control and 95% CI, *P* = 0.00053) and in those individuals not using statins (−79; −130 to −27, ng L^−1^, *P* = 0.003)]. Figure 3 shows that the longer the duration of intervention was, the larger was the group difference in IL-1 Ra. In the fully adjusted analysis (age, gender, body weight, statins, study centre) including three time-points (0, 12 and 18/24 weeks), the intakes of saturated fats [E%, 4.28 (95% CI 0.02; 8.53), *P* = 0.049] and magnesium [mg, −0.23 (−0.41; −0.05), *P* = 0.012] were significantly associated with IL-1 Ra levels (Table 5). Otherwise, no differences were observed in inflammatory markers or HMW adiponectin concentrations between the groups. However, the concentrations of IL-1 beta and IL-10 were low or below functional sensitivity in the majority of the participants (70% and 40% of samples, for IL-1 beta and IL- 10, respectively).

**Table 5:** Association of IL-1 Ra with selected dietary variables^a^

	Estimate (95% CI)	*P*-value
Magnesium (mg)	−0.23 (−0.41 to −0.05)	0.012
SFA (E%)	4.28 (0.02 to 8.53)	0.049
Potassium (mg)	−0.02 (−0.04 to 0.00)	0.073
Fat (E%)	2.04 (−0.49 to 4.57)	0.11
MUFA (E%)	5.13 (−1.14 to 11.40)	0.11
Vitamin C (mg)	−0.16 (−0.35 to 0.04)	0.12
Fibre (g)	−0.95 (−2.39 to 0.50)	0.2
PUFA (E%)	−1.79 (−9.06 to 5.47)	0.63

Data are given as beta estimate with 95% CI.

a After adjustment for age, gender, body weight, statin use and centre (study duration).

## Discussion

In this randomized trial carried out with isocaloric diets, the Healthy Nordic diet, based on Nordic dietary recommendations, did not modify insulin sensitivity and glucose tolerance but resulted in significant between-group changes in non-HDL-C, LDL-C to HDL-C ratio and Apo B to Apo A1 ratio favouring protection from atherosclerosis. Interestingly, as compared to the Healthy diet group, there was a significant increase in IL-1 Ra level with time in the Control diet group. It should be emphasized that our aim was isocaloric study design because we wanted to examine the effect of quality of diet on glucose metabolism, risk factors and inflammation. Concomitant weight loss would make it difficult to interpret the results in this kind of dietary intervention regarding the quality of an experimental diet 13. It can be argued that the results are simply in line to that what can be expected from dietary changes made. However, besides macronutrient and dietary fibre intakes also micronutrients and non-nutrients may have health effects 12, as was the case in the present study, for example, regarding the link between magnesium intake and the change in IL-1 Ra. Thus, obviously, the present study provides novel data on the health effects of Nordic dietary pattern and adds to our current knowledge on the impact of this diet on multitude of metabolic disorders in individuals with features of MetS, not examined previously in detail.

As judged from the dietary records, compliance to the Healthy diet was satisfactory. With regard to phospholipid fatty acid composition, the decrease in dihomo-gamma-linolenic acid proportions and increase in EPA and DHA proportions in the Healthy diet group can be ascribed to higher fish intake 32. Instead, there were no significant differences between the groups for oleic acid content. It is known from controlled feeding trials, where saturated fat (mainly from high-fat dairy products) have been replaced by rapeseed oil in the diet, that the phospholipid proportions of oleic acid, linoleic acid and alpha-linolenic acid increase significantly, whereas the proportions of palmitic acid, palmitoleic acid, dihomo-gamma-linolenic acid and pentadecanoic acid decrease 33.

The lack of effects on glucose metabolism may partly reflect the isocaloric dietary design and that it may be difficult to improve glucose metabolism in established MetS without any concomitant weight loss or more marked changes in diet 23, 29.

The reduction in Apo B to Apo A ratio and non-HDL-C concentration during the healthy Nordic diet indicates the reduction in those lipid fractions that are known to promote atherosclerosis 5, 6. Whilst the non-HDL-C and Apo B lowering effect of the healthy Nordic diet was expected, the trends towards an elevated HDL-C and Apo A1 were less expected, because lowered HDL-C was reported, for example, in the DASH study 34 and after 6-week intervention in the NORDIET study 23, possibly explained by the lower fat content in those diets. However, a diet rich in berries 35, 36, vegetables, fruit and whole grains and foods with low glycaemic index may result in an elevation of HDL-C in the longer term 24, 37. Indeed, Esposito *et al*. also reported an elevation in HDL-C by 0.1 mmol L^−1^ in their study on the long-term effects of a Mediterranean-type diet 14, but there was simultaneous weight reduction during that study 14. According to the present study design, body weight, physical activity and alcohol intake remained essentially unchanged during the trial. Thus, these confounders could not explain the observed results of HDL-C in our study. It is noteworthy that not all studies on the Mediterranean-type diet have reported beneficial effects 38.

The changes in systolic and diastolic blood pressure were not significantly different between the groups. This is in contrast to that seen in the DASH 39 or NORDIET studies 23 with quite similar dietary pattern. In the present study, fat intake was higher as compared to the DASH and NORDIET studies. Furthermore, we achieved only modest changes in self-reported salt intake, and based on 24-h urinary sodium excretion, no reduction in the sodium intake was found in both groups. Perhaps more effort might have been needed in dietary counselling and use of low-salt products.

Amongst the cytokines, IL-1 Ra was markedly and consistently elevated during the Control diet because there was a worsening in the quality of diet. IL-1 Ra is considered as one of the most sensitive markers of inflammation in obesity and MetS 40–44. Furthermore, elevated levels of IL-1 Ra have been shown to predict the onset of type 2 diabetes and progression of MetS to overt type 2 diabetes after adjustment for obesity and hs-CRP 43. The observed elevation of IL-1 Ra may be a compensatory phenomenon 45 to a more atherogenic or proinflammatory composition of the Control diet. Interestingly, IL-1 Ra was associated with the intake of saturated fats (increasing effect) and that of magnesium (decreasing effect), possibly reflecting high intakes of whole-grain products, berries and fruits. Magnesium deficiency may also be directly related to the formation of inflammatory cytokines 46, and in some cohort studies, magnesium intake was inversely associated with systemic inflammation and endothelial dysfunction 47. In the absence of changed CRP and cytokine levels, decreases in IL-1 Ra and liver fat content were recently observed after a diet rich in polyunsaturated compared with saturated fatty acids 48. Furthermore, elevated levels of IL-1 Ra in serum have been related to nonalcoholic steatohepatitis 49. These earlier findings together with our observations suggest that IL-1 Ra is an interesting and also highly sensitive inflammation marker responsive to dietary changes. All other inflammatory markers, including hs-CRP and IL-6, remained unchanged in the present study. This is in contrast to our previous observations where we found reduction in hs-CRP and E-selectin, a marker of endothelial function, during a diet consisting of fatty fish, rye bread and bilberries, thus resembling the present diet 29. The observed difference between these two studies could be explained by the fact that the amounts of rye bread, fatty fish and bilberries consumed by the study individuals were higher in our previous study, whereas no major changes were otherwise allowed, for example, in the fatty acid composition of the experimental diets^29^. In the present study, according to the study design, there was an increase in the intake of saturated fat in the Control diet group, whereas the intake of saturated fat was reduced in the Healthy diet group.

### Strengths and weaknesses

Despite compliance can be considered satisfactory when judging self-reported food records, in this study, the multicentre design including variation in genetic, cultural and dietary aspects could have lowered the chances to observe more pronounced beneficial effects. The larger number of dropouts in the Control diet group could be criticized, but it is explained by the fact that the female study participants were generally aware of healthy dietary principles. Therefore, a number of women randomized into the Control diet group were not willing to continue on the control diet. It can be argued that the variable duration of the study may weaken this study, but this may not be the case as demonstrated in the Fig. 3. One of the strengths of this study is the isocaloric study design achieved for up to 6 months, which made it possible to examine the effects of the dietary composition *as such* on the outcome measurements of interest without concomitant weight changes taking place in many comparative studies. It should also be noticed that the way we performed statistical analyses (i.e. without any exclusions and adjustments) did not change our conclusions.

From a public health point of view, these results are encouraging, as even small reductions in non-HDL-C and LDL-C are considered to have major impact on CVD morbidity and mortality 1, 3, 7. Based on the recent data, non-HDL-C predicts even better the future risk of coronary heart disease than apo B or LDL-C, and the change in non-HDL-C in the present study can be estimated to result in 10% reduction in the coronary heart disease risk 50, 51. Furthermore, a healthy Nordic dietary pattern may have other health benefit as suggested in a recent cohort study in this field 22.

In summary, this study suggests that it is possible to establish a healthy Nordic diet based on the Nordic dietary recommendations and using Nordic and local food items. Although no effect on insulin sensitivity and glucose metabolism was detected, possibly due to the weight-stable conditions, this diet corrected non-HDL-C and LDL-C to HDL-C and corresponding apolipoprotein ratios. A counteracting effect on the inflammation marker IL-1 Ra by the healthy Nordic diet vs. Control diet is an interesting new finding.

## Supporting Information

**Table S1.** Goals of nutrient composition of the study diets.

**Table S2**. Serum phospholipid fatty acid composition (as percentage) at baseline and at the end of the study (mean and SD) by group and the group difference at the end of the study with 95% CI.
