# Supplementary material for: Effects of an isocaloric healthy Nordic diet on insulin sensitivity, lipid profile and inflammation markers in metabolic syndrome – a randomized study (SYSDIET)
Source: J Intern Med. 2013 Mar 2;274(1):52–66. doi: 10.1111/joim.12044 (PMC3749468; doi:10.1111/joim.12044)
Supplement: Supplementary file 1 [file joim0274-0052-SD1.docx]

Supplement table 1. Goals of nutrient composition of the study diets.

| Nutrient | Healthy diet | Control diet |
| --- | --- | --- |
| Carbohydrate (E%) | 45-52 | 45-47 |
| Sucrose (E%) | ≤ 10, max. 50 g/day | no restrictions |
| Fibre (g) | ≥ 35 g or 4g/MJ | 15-20 |
| Protein (E%) | 18-20 | 18-20 |
| Fat (E%) | 30-35 | 35 |
| Saturated (E%) | < 10 | 15 |
| Monounsaturated + polyunsaturated (E%) | Minimum 2/3 of total fat intake | 15 5 |
| Salt (g) | 6 g for women, 7g for men | ≤ 10 |

**Supplemental table 2**. Serum phospholipid fatty acid composition (as percentage) at baseline and at the end of the study (mean and SD) by group and the group difference at the end of the study with 95 % CI.

|  | **Healthy diet (n=85-95)** | | **Control diet (n=63-73)** | |  |  |
| --- | --- | --- | --- | --- | --- | --- |
|  | **Baseline** | **End** | **Baseline** | **End** | **Estimate (95% CI)** | **P- value** |
| **Myristic (14:0)** | 0.44 (0.09) | 0.43 (0.09) | 0.43 (0.10) | 0.45 (0.09) | -0.03 (-0.05 to 0.00) | 0.08 |
| **Pentadecanoic (15:0)** | 0.25 (0.07) | 0.23 (0.07) | 0.25 (0.07) | 0.26 (0.06) | -0.02 (-0.03 to -0.01) | 0.00024 |
| **Palmitic (16:0)** | 31.09 (1.16) | 30.88 (1.24) | 31.52 (1.19) | 31.33 (1.15) | -0.06 (-0.37 to 0.24) | 0.69 |
| **Palmitoleic (16:1)** | 0.72 (0.25) | 0.68 (0.26) | 0.79 (0.27) | 0.77 (0.26) | -0.02 (-0.08 to 0.04) | 0.47 |
| **Heptadecanoic (17:0)** | 0.37 (0.07) | 0.36 (0.07) | 0.37 (0.05) | 0.37 (0.05) | -0.01 (-0.02 to 0.01) | 0.26 |
| **Stearic (18:0)** | 14.83 (1.06) | 14.81 (1.13) | 14.30 (1.00) | 14.50 (0.97) | -0.15 (-0.40 to 0.10) | 0.24 |
| **Oleic (18:1)** | 11.43 (1.07) | 11.19 (1.07) | 11.94 (1.24) | 12.01 (1.01) | -0.35 (-0.68 to -0.02) | 0.039 |
| **Linoleic (18:2)** | 19.19 (2.54) | 19.60 (2.24) | 19.02 (2.08) | 19.67 (2.58) | -0.10 (-0.71 to 0.51) | 0.74 |
| **Gamma-linolenic (18:3(n-6))** | 0.09 (0.04) | 0.09 (0.04) | 0.10 (0.04) | 0.10 (0.04) | -0.00 (-0.01 to 0.01) | 0.91 |
| **Alpha-linolenic (18:3(n-3))** | 0.28 (0.10) | 0.32 (0.13) | 0.26 (0.10) | 0.27 (0.10) | 0.03 (-0.00 to 0.06) | 0.076 |
| **Di-homo-gamma-linolenic (20:3(n-6))** | 3.16 (0.69) | 2.93 (0.63) | 3.49 (0.58) | 3.62 (0.63) | -0.30 (-0.46 to -0.15) | 0.00021 |
| **Aracidonic (20:4(n-6))** | 9.39 (1.68) | 8.96 (1.75) | 9.68 (1.63) | 9.48 (1.74) | -0.27 (-0.61 to 0.07) | 0.12 |
| **EPA (20:5)** | 2.46 (1.29) | 2.86 (1.24) | 2.02 (0.73) | 1.71 (0.57) | 0.68 (0.35 to 1.00) | 7.50E-05 |
| **DPA (22:5)** | 1.10 (0.21) | 1.09 (0.20) | 1.05 (0.20) | 1.03 (0.20) | 0.01 (-0.03 to 0.05) | 0.67 |
| **DHA (22:6)** | 5.22 (1.28) | 5.57 (1.04) | 4.75 (0.90) | 4.42 (0.91) | 0.63 (0.36 to 0.90) | 9.70E-06 |
